# *In Vitro* Antioxidant Activity and Hepatoprotective Effects of *Lentinula edodes* against Paracetamol-Induced Hepatotoxicity

**DOI:** 10.3390/molecules15064478

**Published:** 2010-06-23

**Authors:** Sreenivasan Sasidharan, Sugumaran Aravindran, Lachimanan Yoga Latha, Ratnasamy Vijenthi, Dharmaraj Saravanan, Santhanam Amutha

**Affiliations:** 1 Institute for Research in Molecular Medicine (INFORMM), Universiti Sains Malaysia, 11800, Pulau Pinang, Malaysia; 2 Department of Biotechnology, Faculty of Applied Sciences, AIMST University, Jalan Bedong-Semeling, Batu 3 ½, Bukit Air Nasi, Bedong 08100, Kedah, Malaysia; 3 School of Biological Sciences, Universiti Sains Malaysia, 11800, Pulau Pinang, Malaysia; 4 Centre for Drug Research, Universiti Sains Malaysia, 11800, Pulau Pinang, Malaysia; 5 School of Biotechnology, Madurai Kamaraj University, Palkalai Nagar, Madurai, India

**Keywords:** *Lentinula edodes*, hepatoprotective activity, antioxidant activity, liver marker enzymes

## Abstract

*Background*: The objective of this study was to investigate the antioxidant and hepatoprotective effects of methanolic extracts of *L. edodes* and the determination of their total phenolics content. *Results*: The amount of total phenolics was estimated to be 70.83 mg Gallic Acid Equivalent (GAE) per gram of dry extract. The antioxidant activity of the *L. edodes* extract was 39.0% at a concentration of 1 mg/mL and was also concentration dependant, with an EC_50_ value of 4.4 mg/mL. Different groups of animals (Wister albino mice) were administered paracetamol (1 g/kg, p.o.). *L. edodes* extract at a dose of 200 mg/kg was administered to the paracetamol treated mice for seven days. The effects of *L. edodes* extract on serum transaminases (SGOT, SGPT), alkaline phosphatase (ALP) and bilirubin were measured in the paracetamol-induced hepatotoxic mice. *L. edodes* extract produced significant (*p* < 0.05) hepatoprotective effects by decreasing the activity of serum enzymes and bilirubin. *Conclusion*: From these results, it was suggested that *L. edodes* extract could perhaps protect liver cells from paracetamol-induced liver damage by its antioxidative effect on hepatocytes, hence diminishing or eliminating the harmful effects of toxic metabolites of paracetamol.

## 1. Introduction

Recently, edible basidiomycetes have been considered by scientists as potential natural resources to develop antioxidant compounds. This is a result of the fact that they are good sources of secondary metabolites, vitamins, minerals, proteins and carbohydrates, as well as being high in fiber and low in fat [1]. Since primordial days, mushrooms have been always consumed in human’s daily diet as an additional supplementary item. Mushrooms are fleshy, spore-bearing fruiting bodies of a fungus, typically produced above ground on soil or on its food source. Mushroom consumption is growing worldwide due to the influence of Oriental culture, studies on their nutritional values and pharmacological properties [2]. Most mushrooms contain vitamins, particularly niacin, thiamine, riboflavin, biotin and vitamin C. Mushrooms also contain a wide variety of bioactive molecules including terpenoids, steroids, phenols, nucleotides and the glycoprotein derivatives and polysaccharides [3]. In addition to their nutritional value mushrooms are claimed to exhibit antitumour, antiviral, antibiotic, anti-inflammatory, hypoglycaemic, hypocholesterolemic and hypotensive activities [4,5].

Oxidative damage caused by free radicals may be related to aging and diseases, such as atherosclerosis, diabetes, cancer and cirrhosis [6]. Antioxidant compounds reduce the action of reactive oxygen species (ROS) in damaged tissues during the recovery process [7]. The search for new bioactive products with antioxidant activity has lead to the present study, whose aims were to investigate the antioxidant activity and the effects of the methanol extracts of *L. edode* on liver function markers in the serum, as well as on hepatic histopathology of the mice liver showing paracetamol induced hepatotoxicity.

## 2. Results and Discussion

### 2.1. DPPH scavenging assay and EC_50_ determination

**Figure 1:**
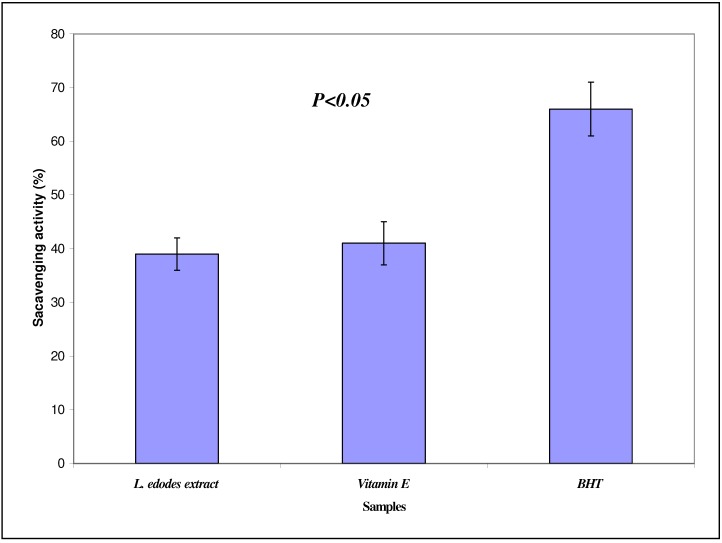
Scavenging effect (%) of crude extract of *Lentinula edodes*, and the known antioxidants BHT and vitamin E at 1.0 mg/mL.

The antioxidant potential of *L. edodes* methanol extract was investigated in the search for new bioactive compounds from natural resources. The measured DPPH radical scavenging activity is depicted in Figure 1. The free radical scavenging activities were 39.0%, 41.0% and 66.00% for the *L. edodes* extract, vitamin E and BHT, respectively. The EC_50_ value is 4.4 mg/mL (y = 11.7x - 1.693, R^2^ = 0.988) which is the concentration of the crude extract that decreases the initial DPPH radical concentration by 50%. Effectiveness of antioxidant properties is inversely correlated with EC_50_ values. Lee *et al.* [8] reported that if the EC_50_ value of an extract is less than 10 mg/mL, it indicates that the extract is an effective antioxidant. In this study, the EC_50_ value of the shiitake mushroom extract was less than 10 mg/mL, and this indicates that the extract was an effective antioxidant. In this study Soxhlet method was use for the extraction. Similar method was also reported by Ramesh *et al.* [9]. They used six medicinal mushroom extracts extracted by soaking method for 24 h to evaluate antimicrobial properties and antioxidant activity.

However the antioxidant activity of the extract was lower compared to BHT and vitamin E, the reference antioxidants. This could be due to the lack of DPPH sensitive bioactive compounds in the crude extract. Other anti-oxidant assays need to be undertaken to verify this point. Other possible explanation would be the presence of the actual bioactive compound in a small concentration in the crude extract. Further work need to be carried out on the isolation and purification of the bioactive components with good antioxidant activity from the crude extract of mushroom. Similar results were also reported by Kitzberger *et al.* [10]. They used extracts of *L. edodes* obtained by organic solvents and supercritical fluids to test the antioxidant activity. Their results indicate that the fractions obtained using CO_2_ and ethanol as co-solvents at 40 ºC with pressure of 20 MPa had similar antioxidant results to the composition obtained with 15% ethanol and dichloromethane using the low-pressure technique. Cheung and Cheung [11], also reported the antioxidant activity of *L. edodes* against lipid peroxidation. They found that the low molecular weight sub-fraction of the water extract of *L. edodes* had the highest antioxidant activity against lipid peroxidation of rat brain homogenate, with IC_50_ values of 1.05 mg/mL.

In addition, other mushrooms have also been reported to possess antioxidant activity. Wong and Chye [12] reported the antioxidant activity of *Pleurotus porrigens, Hygrocybe conica, Xerula furfuracea (Rooted oude), Schizophyllum Commune, Polyporus tenuiculus (Pore fungus)* and *Pleurotus florida*. Based on the results they obtained, petroleum ether (PE) and methanolic extracts from these edible wild mushrooms were effective in DPPH radical scavenging and metal chelating ability. PE extracts were more effective than methanolic extracts in antioxidant activity using the DPPH, whereas methanolic extracts were more effective in reducing power and metal chelating ability.

### 2.2. Total phenolic content

It has been reported that the antioxidant activity of shiitake mushroom was well correlated with the content of their phenolic compounds [13]. The total phenolic content measured in this study was 70.83 mg Gallic Acid Equivalents (GAEs) per gram of dry mushroom.

### 2.3. Hepatoprotective activity of L. edodes methanolic extract

**Figure 2:**
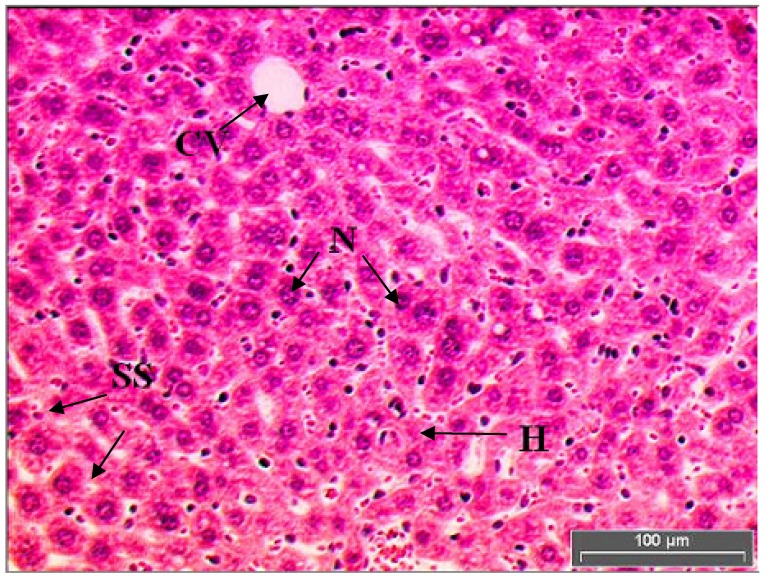
Light microphotographs of haematoxylin and eosin stained sections of the formalin fixed liver cell of normal mice. Liver cells of Group 1 mice (normal) have hepatic cells with well-preserved cytoplasm, prominent nucleus and nucleolus. (H, hepatocytes; N, nucleus; SS, sinusoid; CV, central vein).

Figure 2 shows the liver tissue of mice which received 1 mL/kg of saline and free access to pellets. Control group showed a normal liver architecture of hepatocytes where they were well arranged without any alteration at central and portal veins.

These normal hepatocytes are polyhedral in shape with defined cell lining in the liver tissue. The cytoplasm is well preserved with prominent nucleus and nucleolus (cf. Figure 4). Hepatocytes make up 70–80% of the cytoplasmic mass of the liver. These cells are involved in protein synthesis, protein storage and transformation of carbohydrates. Other roles for these cells are synthesis of cholesterol, bile salts and phospholipids, as well as detoxification, modification and excretion of exogenous and endogenous substances [14].

**Figure 3:**
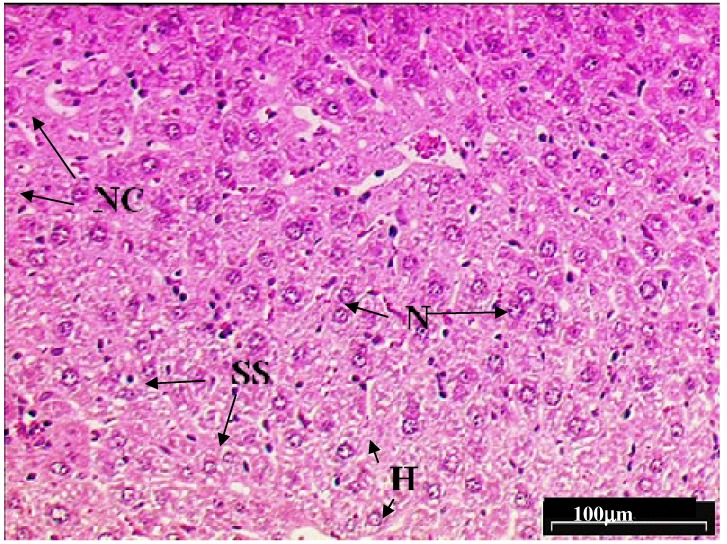
Light microphotographs of haematoxylin and eosin stained sections of the formalin fixed liver cell of mice exposed to paracetamol. Liver cells of Group II mice (exposed to paracetamol) revealed extensive fatty changes, characterized by the disruption of the lattice nature of the hepatocyte, damaged hepatic sinusoids and necrosis. Presences of reticular sides are visible and nucleuses of two to three are joined together. (H, hepatocytes; N, nucleus; SS, sinusoid; NC, necrosis).

**Figure 4:**
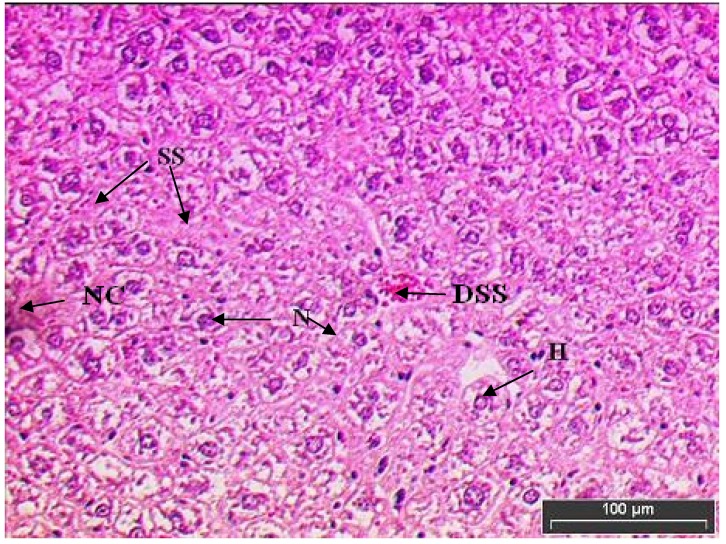
Light microphotographs of haematoxylin and eosin stained sections of the formalin fixed liver cells of mice treated with mushroom extract. Liver cells of Group III mice (exposed to paracetamol and mushroom extract), only minimal disruption of the hepatic cellular structure was observed. Nucleases are at recovery stages and absence of joined nuclease. Sinusoids are slowly recovering and there presence of dilated sinusoid filled with red blood cells. (H, hepatocytes; N, nucleus; SS, sinusoid; NC, necrosis; DSS, dilated sinusoid filled with red blood cells).

Figure 3 shows mice liver tissue damage induced with paracetamol (1.0 g/kg paracetamol orally × 7 days). Toxic effect such as liver damage, haemolytic anaemia, oxidative damage to the red blood cells and bleeding tendencies due to over dosage of paracetamol was noted. Marked distorted hepatocyte structure was observed in the liver of mice induced with paracetamol alone. The liver showed severe toxicity characterized by inflammatory cell collection and scattered inflammation. The cell lining of the hepatocytes are not visible after being exposed to high toxicity. Besides that, the occurrence of focal necrosis, unnatural death, the progression of living tissue around the cell body and dented hepatic sinusoids of cells were observed. Clumping of two or more nucleus at one position, which can lead to carcinogenesis, was noted (Figure 4).

Figure 4 shows the mice liver tissue induced with paracetamol and treated with *L. edodes* extract. *L. edodes* is highly known for its medicinal value as an antioxidant agent that prevents free radicals produced by paracetamol toxicity. In this study, mice were given oral paracetamol 1 g/kg to induce hepatotoxicity and this was challenged by 200 mg/kg of mushroom extract 3 h after the administration of paracetamol. The treatment was continued for seven days. Only minimal disruption of the structure of hepatocytes was noted in liver tissue of mice exposed to paracetamol and *L. edodes* extract.

The liver tissue of mice treated with *L. edodes* extract displayed cell recovery compared to the mice induced with paracetamol alone (Figure 4). Hepatocytes were being transformed to normal polyhedral shape with some cell lining observed. Nucleuses are slowly improving and clumping of nucleus is not seen.

### 2.4. Liver marker enzymes and bilirubin content

The effect of *L. edodes* extract on liver marker enzymes and serum bilirubin content are given in Table 1.

Acute paracetamol administration significantly increased the level of liver injury marker enzymes like SGOT, SGPT, and ALP (The liver marker enzymes were 118.4 ± 11.3 IU/L, 81.2 ± 5.3 IU/L and 129.3 ± 7.3 Units/L for the SGOT, SGPT and ALP, respectively). A similar experimental procedure also used by other researchers to report the hepatoprotective effect of natural products. Kumar *et al.* [15] reported on the hepatoprotective effects of some edible mushrooms using paracetamol (APAP)- induced liver injury in the rat as a model. They reported the degree of protection was measured by using biochemical parameters like serum glutamate oxaloacetate transaminase (SGOT), serum glutamate pyruvate transaminase (SGPT), alkaline phosphatase (ALP), bilirubin (BRN), and total protein (TP). Administration of *L. edodes* extract (200 mg/kg body weight) decreased the damaging effect as evidenced from the low levels of these enzymes in the serum. Our studies showed that the treatment with *L. edodes* extract for seven days offers considerable protection to liver as evidenced from the levels of biochemical parameters (The liver marker enzymes were 55.13 ± 6.3 IU/L, 30.6 ± 4.7 IU/L and 58.4 ± 6.2 Units/L for the SGOT, SGPT and ALP, respectively), which was supported by the limited extent of histological changes. The paracetamol administered group showed elevated increase in serum bilirubin (9.9 ± 3.4 mg/L). Treatment of *L. edodes* extract restored the altered bilirubin to the normal levels (2.8 ± 2.4 mg/mL).

Bilirubin concentration has been used to evaluate chemically induced hepatic injury. Besides various normal functions, liver excretes the breakdown product of hemoglobin namely bilirubin into bile. It is well known that necrotizing agents like paracetamol produce sufficient injury to hepatic parenchyma to cause large increases in bilirubin content [16]. *L. edodes* extract prevented severity of liver damage caused by paracetamol as evidenced by the low level of bilirubin in the serum. Similar results were also reported by Jayakumar *et al.* [17]. They used oyster mushroom (*Pleurotus ostreatus*) extracts on CCl_4_-induced liver damage in male Wistar rats. They reported that when rats with CCl_4_- induced hepatotoxicity were treated with the extract of *P. ostreatus*, the serum SGOT, SGPT and SALP levels reverted to near normal.

Vitamin E used as reference in scavenging effect tests also reported to have stronger hepatoprotective effect. A previous study by Uboh *et al.* [18] showed that the vitamin E possessed hepatoprotective effects. They reported that their study indicated that the percentage decreases in the activities of serum enzymes and total serum bilirubin, as well as percentage increase in total serum protein levels, in both male and female test rats treated with α-tocopherol were significantly higher (P < 0.05) compared to the respective percentage changes obtained for both sexes of rats treated with retinol. These results suggested that although retinol and α-tocopherol may enhance recovery from hepatotoxicity associated with exposure to gasoline vapors in male and female rats, the hepatoprotective potency of α-tocopherol tends to be higher than that of retinol. Moreover, *L. edodes* contains polysaccharide fractions has been tested previously and was reported to have a significant hapotoprotective activity [19]. It is possible that this compound was mainly responsible for the observed hapotoprotective activity effects seen in this study.

## 3. Experimental

### 3.1. Mushroom material

Fresh fruiting bodies of *L. edodes* (Shiitake mushroom) were purchased from a local wet market at Bedong, Kedah, Malaysia in June 2008. The *L. edodes* fruiting bodies were washed with running water followed by distilled water to remove residual compost on the sample surface. Consequently mushrooms were left to dry in the oven at 60 °C for 7 days to ensure complete dryness. The sample was then ground using a blender (0.5 mm sieve) and stored for further usage.

### 3.2. Extraction of L. Edodes

The fine dried mushroom powdered sample (60 g) was extracted with 95% methanol (b.p 64–66 ºC, 300 mL) for 48 h using a Soxhlet extractor. Subsequently, the methanol extract was evaporated with a rotary evaporator at 50 ºC to dryness and stored for further usage.

### 3.3. Antioxidant activity assays

2,2-Diphenyl-1-picrylhydrazyl radical (DPPH) scavenging assay quantitative measurement of radical scavenging properties was carried out in a universal bottle. The reaction mixture contained test sample (50 µL) ranging in concentration from 0.5 mg/mL to 6 mg/mL and 0.004 w/v % DPPH solution in methanol (5 mL, 80% v/v). The mixture without test sample was used as blank and spiked with 50 µL of blank methanol. The commercial antioxidant butylated hydroxytoluene (BHT, Sigma) was used for comparison or as a positive control. Discoloration was measured at 517 nm after being incubated for 30 min. Measurements were taken in triplicates. DPPH radical’s concentration was calculated using the following equation:

DPPH scavenging effect (%) = A_o_ – A_1_ / A_o_ X100

where A_o_ is control absorbance and A_1_ is the absorbance in the presence of the sample (extract of the mushroom) [20]. The actual decrease in the absorbance induced by the test sample was compared with the positive controls.

### 3.4. EC_50_ determination

The extract concentration that exhibited 50% inhibition (EC_50_) is calculated from the graph of RSA percentage against extract concentrations. BHT and α-tocopherol were used as standards. The EC_50_ was defined as the amount of the sample that is sufficient to elicit 50% reduction of the initial DPPH concentration. This was calculated from the linear regression plots of test extract (mg/mL) concentration against the mean percentage of antioxidant activity obtained from three replicate tests [21].

### 3.5. Determination of total phenolic content

Total phenolic content was determined by the Folin–Ciocalteu reagent assay. The total phenolic concentration in the mushroom extract was expressed as gallic acid equivalents (GAEs) and was measured according to the method described by Singleton and Rossi [10] with slight modifications. The sample (l mL) was mixed with Folin–Ciocalteu phenol reagent (Sigma, 1 mL). The solution was then allowed to stand for 3 min at 25 ºC before adding saturated sodium carbonate solution (Na_2_CO_3_~35%, 1 mL). The final volume was made up to 10 mL with deionized water. The reaction was kept in a dark place for 120 min and the absorbance was read at 765 nm. Gallic acid was used as standard for the calibration curve. The total phenolic content was expressed as mg gallic acid equivalents (GAE) per gram of sample [22].

### 3.6. Hepatoprotective activity of L. edodes extract

#### 3.6.1. Animals

Wister albino mice of either sex were used to study the hepatoprotective activity of the *L. edodes* extract. The Institution Animal Ethics Committee of AIMST University has approved the animal study for this project. The animals were kept at 27 ± 2 ºC, relative humidity 44–56% and light and dark cycles of 10 and 14 h respectively, for a week before and during the experiments. Animals were provided with standard diet (Lipton, India) and water *ad libitum*. The food was withdrawn 18–24 h before starting the experiment. All experiments were performed in the morning according to current guidelines for the care of the laboratory animals and the ethical guidelines for the investigation of experimental pain in conscious animals [23].

#### 3.6.2. Paracetamol dose regimen

Paracetamol tablets were obtained from a nearby clinic. Each tablet contains 500 mg of paracetamol. The dose administered to the mice was set as 1 g/kg. The paracetamol was made into fine powder using a mortar and pestle. The powdered paracetamol was suspended in saline and was administered according to the body weight of mice.

#### 3.6.3. Grouping of mice and treatments

Eighteen mice (25–30 g) were randomly divided into three groups and each group consists of 6 mice (Table 2). The first group received 1 mL/kg of saline (control). Group II was given paracetamol (1.0 g/kg) orally and group III received orally both 1.0 g/kg paracetamol [24] and 200 mg/kg of *L. edodes* extract respectively. Extract was administered three hours after the administration of paracetamol. Paracetamol 1g/kg was given to mice to induce hepatotoxicity. The treatments were continued for seven days and on the Eighth day of the experiment; all animals were anesthetized and dissected [25].

#### 3.6.4. Sacrifice and organ harvesting

The liver was removed carefully after euthanizing and killing the animals by cervical dislocation. The livers were fixed in 10% buffered formalin. After fixation, the livers were dehydrated in a graded series of alcohol, cleared in xylene and embedded in paraffin wax. Multiple 5 µm sections from each block were mounted on slides and stained with hematoxylin and eosin.

#### 3.6.5. Biochemical parameters

The mice of each group were anaesthetized with ether, and blood was collected directly from the heart. It was centrifuged at 2,000 g for 10 min at 4 ºC to separate the serum and kept at 4 ºC to assay the activities of serum enzymes. Glutamate oxaloacetate transaminase (SGOT) and glutamate pyruvate transaminase (SGPT) were determined by the method of Reitman and Frankel as described by Bergrneyer and Bernt [26]. Alkaline phosphatase (ALP) was estimated according to Kind and King [27]. Serum bilirubin level was estimated according to Malloy and Evelyn [28].

### 3.7. Statistical analysis

All values are mean±S.E.M. obtained from six animals. For statistical analysis, one-way ANOVA with Duncan’s variance (SPSS 15) was used to compare the groups. In all the cases a difference was considered significant when *p* <0.05.

## 4. Conclusions

These findings show that the *L. edodes* extract possesses antioxidant activity with hepatoprotective properties. Since the *L. edodes* is a very popular food in Asia and the raw materials can be stably supplied by cultivation of the mycelia, the extract is a promising candidate for use as an antioxidant and hepatoprotective agent.

## Figures and Tables

**Table 1:** Effect of *L. edodes* extract on liver marker enzymes and serum bilirubin content.

Parameters	Control	Induced	Treatment
SGOT (IU/L)	45.1 ± 5.4	118.4 ± 11.3**	55.13± 6.3*
SGPT (IU/L)	27.3 ± 4.1	81.2 ± 5.3**	30.6 ± 4.7*
ALP (Units/L)	51.1 ± 4.9	129.3 ± 7.3**	58.4 ± 6.2*
Bilirubin (mg/L)	1.4 ± 0.4	9.9 ± 3.4**	2.8 ± 2.4*

Results are expressed as mean ± S.E.M. of six animals; * Statistically significant compared to induced animals (*p* < 0.05); ** Statistically significant to control animals (*p* < 0.05).

**Table 2:** Grouping of mice and treatment administered.

GROUPS	TREATMENTS
Control	1mL/kg of saline of per body weight
Induced	1g/kg of paracetamol per body weight
Treatment	1g/kg of paracetamol + 200 mg/kg of extract per body weight

18 adult mice were divided into 3 groups (n = 6)

## References

[1] Manzi L., Marconi G.S., Vivanti V., Pizzoferrato L. (1999). Nutrients in edible mushrooms: an inter- species comparative study. Food Chem..

[2] Yang J.H., Linb H.C., Maub J.L. (2002). Antioxidant properties of several commercial mushrooms. Food Chem..

[3] Borchers A.T., Stern J.S., Hackman R.M., Keen C.L., Gershwin M.E. (1999). Mushrooms, tumors, and immunity. Proc. Soc. Exp. Biol. Med..

[4] Hobbs C.H., Michael M. (2003). An Exploration of Tradition, Healing and Culture. Medicinal Mushrooms.

[5] Ooio V.E., Liu F. (1999). A review of pharmacology activities of mushroom polysaccharides. Int. J. Med. Mushrooms.

[6] Halliwell B., Gutteridge J.M.C. (1984). Oxygen toxicity, oxygen radicals, transition metals and disease. Biochem. J..

[7] Barros L., Baptista P. (2007a). Ferreira ICFR: Effect of *Lactarius piperatus* fruiting body maturity stage on antioxidant activity measured by several biochemical assays. Food. Chem. Toxicol..

[8] Lee Y.L., Jian S.Y., Lian P.Y., Mau J.L. (2008). Antioxidant properties of extracts from a white mutant of the mushroom *Hypsizigus marmoreus*. J. Food Compos. Anal..

[9] Ramesh Ch., Pattar M.G. (2010). Antimicrobial properties, antioxidant activity and bioactive compounds from six wild edible mushrooms of western ghats of Karnataka, India. Phcog. Res..

[10] Kitzberger C.S.G., Smânia A., Pedrosa R.C., Ferreira S.R.S. (2007). Antioxidant and antimicrobial activities of shiitake (*Lentinula edodes*) extracts obtained by organic solvents and supercritical fluids. J. Food Eng..

[11] Cheung L.M., Cheung P.C.K. (2005). Mushroom extracts with antioxidant activity against lipid oxidation. Food Chem..

[12] Wong J.Y., Chye F.Y. (2009). Antioxidant properties of selected tropical wild edible mushrooms. J. Food Compos. Anal..

[13] Velioglu Y.S., Mazza G., Gao L., Oomah B.D. (1998). Antioxidant activity and total phenolics in selected fruits, vegetables, and grain products. J. Agric. Food Chem..

[14] Hamel J.C., Goujon M., Aldigier C.E., Touchard G., Cogne M. (2006). The survival of hematopoietic cells and hepatocytes in mice. J. Blood.

[15] Kumar G., Banu G.S., Pappa P.V., Sundararajan M., Pandian M.R. (2004). Hepatoprotective activity of *Trianthema portulacastrum* L. against paracetamol and thioacetamide intoxication in albino rats. J. Ethnopharmacol..

[16] Plaa G.L., Hewitt W.R., Hayes A.W. (1982). Detection and evaluation of chemical induced liver injury. Principles and Methods of Toxicology.

[17] Jayakumar T., Ramesh E., Geraldine P. (2006). Antioxidant activity of the oyster mushroom, *Pleurotus ostreatus*, on CCl_4_-induced liver injury in rats. Food Chem. Toxicol..

[18] Uboh F.E., Ebong P.E., Umoh I.B. (2009). Comparative hepatoprotective effect of vitamins A and E against gasoline vapor toxicity in male and female rats. Gastroenterol Res..

[19] Wasser S.P. (2005). Shiitake (*Lentinus edodes*). Encyclopedia of Dietry Supplement.

[20] Sasidharan S., Darah I., Jain N.M.K.M. (2007). Free radical Scavenging Activity and Total Phenolic Compounds of *Gracilaria changii*. Int. J. Nat. Eng. Sci..

[21] Aderogba M.A., Okoh E.K., Adelanwa T.A., Obuotor E.M. (2004). Antioxidant properties of the Nigerian *Piliostigma* species. J. Biol. Sci..

[22] Singleton V.L., Rossi J.A. (1965). Colorimetric of total phenolics with phosphomolybdic- phosphotungstic acid reagents. Am. J. Enol. Viticult..

[23] Zimmerman M. (1983). Ethical guidelines for investigation of experimental pain in conscious animal. Pain.

[24] Rao P.G., Rao G., Ramnarayan K., Srinivasan K.K. (1993). Effect of hepatogard on paracetamol- induced liver injury in male albino rats. Indian Drugs.

[25] da Rocha R.P., de Miranda Paquola A.C., do Valle Marques M., Menck C.F.M., Galhardo R.S. (2008). Characterization of the SOS Regulon of *Caulobacter crescentus*. J. Bacteriol..

[26] Bergmeyer H.U., Bernt E., Bergmeyer H.U. (1974). Colorimetric assay of Reitman and Frankel. Methods of Enzymatic Analysis.

[27] Kind P.R.N., King E.J. (1954). Estimation of plasma phosphatase by determination of hydrolysed phenol with antipyrin. J. Clin. Pathol..

[28] Malloy H.T., Evelyn K.A. (1937). The determination of bilirubin with the photochemical colorimeter. J. Biol. Chem..

